# Supplementary material for: Identification of CFAP52 as a novel diagnostic target of male infertility with defects of sperm head-tail connection and flagella development
Source: eLife. 2023 Dec 21;12:RP92769. doi: 10.7554/eLife.92769 (PMC10735225; doi:10.7554/eLife.92769)
Supplement: Figure 3—source data 1. [file elife-92769-fig3-data1.zip › Figure 3-source data 1/Figure 3-source data 1/Figure 3-source data 1.docx]

**Figure 3—source data 1.** Primers for Sanger sequencing and Minigene.

| **Variant** | **Sequence (5’→3’)** |
| --- | --- |
| c.203G>T | AAAAAAAAGAAAGAAAAAAGA |
|  | ATAGAAGGGAACACAGACACT |
| c.1128G>A | ATTATTTCACTTGGTTTTGCG |
|  | CACTCCTCGTTGTTCCTCTTC |
| RT-PCR for *Cfap52* | TCACAAGGGCAAAATCGAAGC |
|  | CTCATCTCGGCACCTAGAGAA |
| RT-PCR for *Gapdh* | AGGTCGGTGTGAACGGATTTG |
|  | TGTAGACCATGTAGTTGAGGTCA |
| RT-PCR between SD6 and SA2 in plasmid | TCTGAGTCACCTGGACAACC |
|  | ATCTCAGTGGTATTTGTGAGC |
