# Supplementary material for: Identification of CFAP52 as a novel diagnostic target of male infertility with defects of sperm head-tail connection and flagella development
Source: eLife. 2023 Dec 21;12:RP92769. doi: 10.7554/eLife.92769 (PMC10735225; doi:10.7554/eLife.92769)
Supplement: Figure 4—source data 1. [file elife-92769-fig4-data1.zip › Figure 4-source data 1/Figure 4-source data 1/Figure 4-source data 1.docx]

**Figure 4—source data 1.** Primers for *Cfap52*-KO mouse genotyping.

| **Primer** | **Sequence (5’→3’)** | **Band size** |
| --- | --- | --- |
| F1 | CTCAACATGCAAGCAGGAATCTCTC | WT: 4210 bp  KO: 377 bp |
| R1 | CTGTGCATTGAGAGGACATGGAA |  |
| F2 | CCACCACCCAGATACTTACATGTCT | WT: 466 bp  KO: 0 bp |
| R2 | TTCAGTTGTTCAGCTGTCACTGGG |  |
