# Supplementary figures and images for: Identification of CFAP52 as a novel diagnostic target of male infertility with defects of sperm head-tail connection and flagella development
Source: eLife. 2023 Dec 21;12:RP92769. doi: 10.7554/eLife.92769 (PMC10735225; doi:10.7554/eLife.92769)

Figure 4C


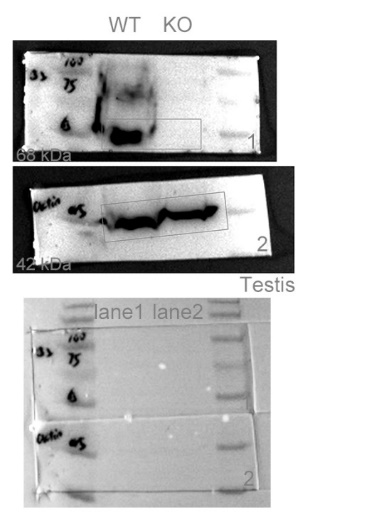

Supplement: Figure 4—source data 2. [file elife-92769-fig4-data2.zip › Figure 4-source data 2/Figure 4-source data 2/Figure 4-source data 2/Figure 4C-labeled.docx]

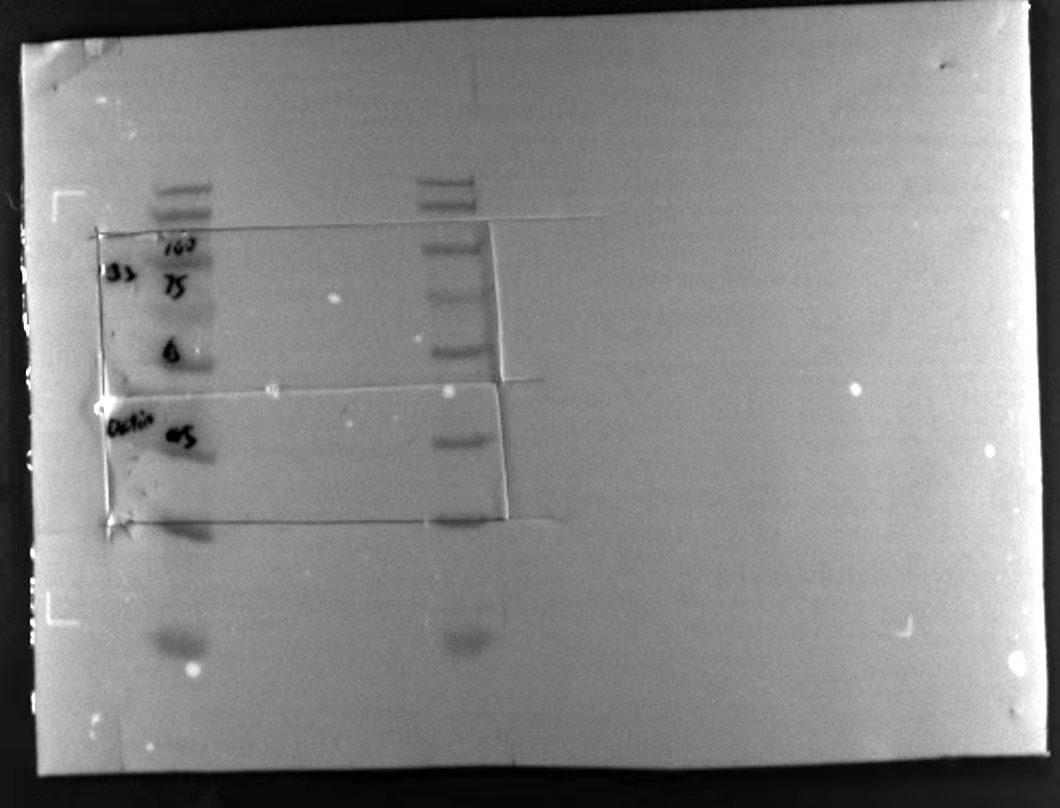

Supplement: Figure 4—source data 2. [file elife-92769-fig4-data2.zip › Figure 4-source data 2/Figure 4-source data 2/Figure 4-source data 2/Raw images/1.jpg]

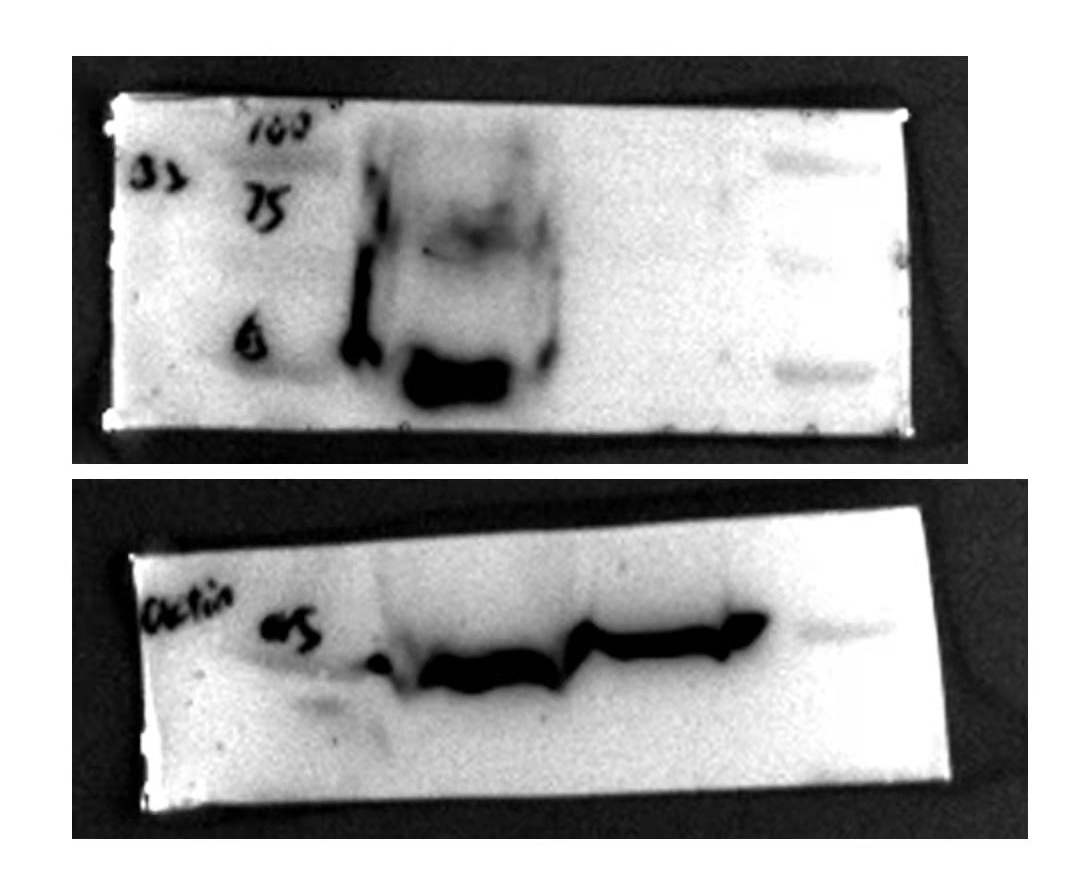

Supplement: Figure 4—source data 2. [file elife-92769-fig4-data2.zip › Figure 4-source data 2/Figure 4-source data 2/Figure 4-source data 2/Raw images/2.jpg]

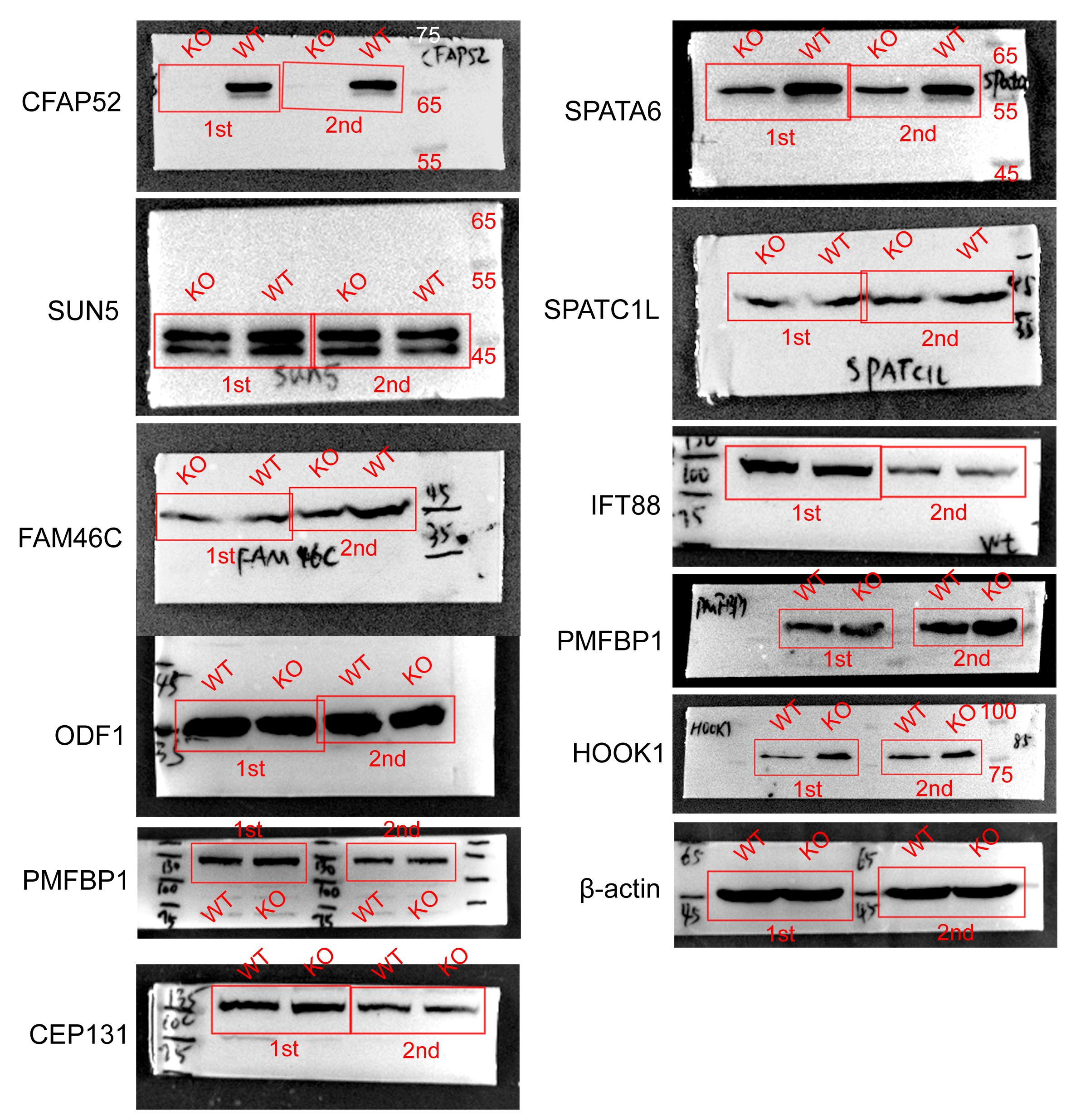

Supplement: Figure 6—source data 2. [file elife-92769-fig6-data2.zip › Figure 6-source data 2/Figure 6-source data 2/Figure 6-source data 2/Blots for Figure 6A.png]

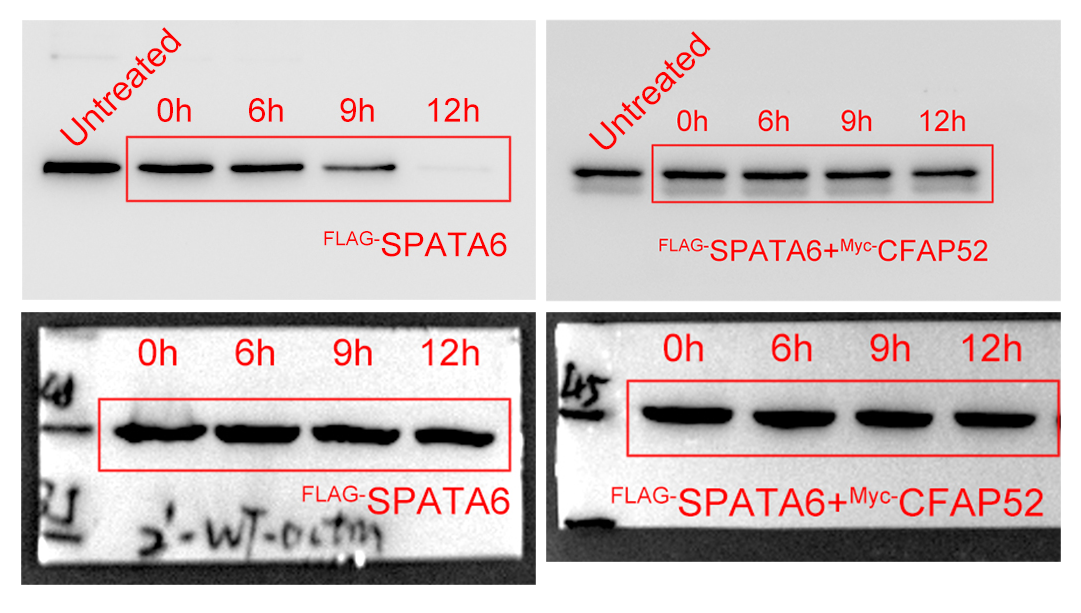

Supplement: Figure 6—source data 2. [file elife-92769-fig6-data2.zip › Figure 6-source data 2/Figure 6-source data 2/Figure 6-source data 2/Blots for Figure 6E, F.tif]

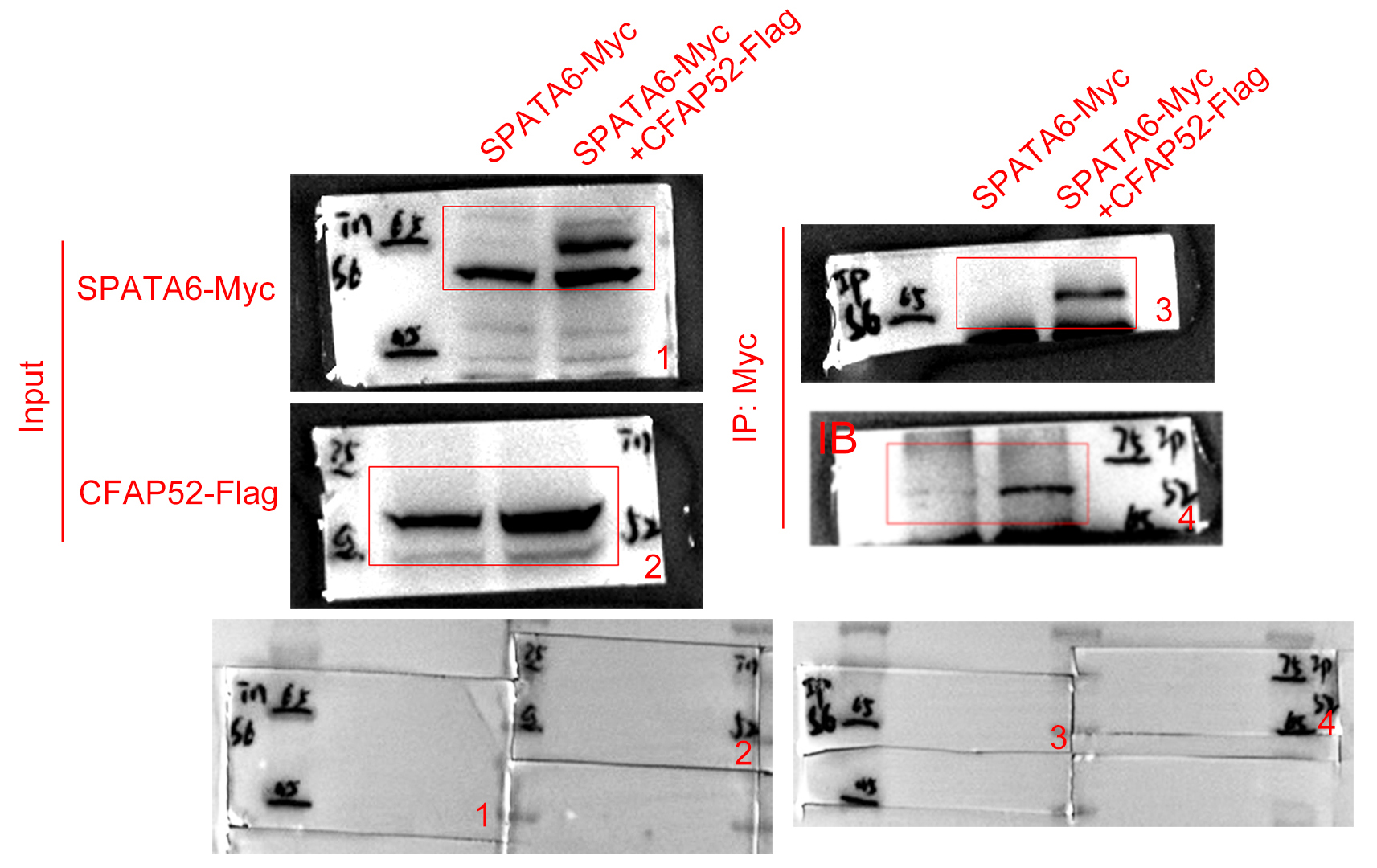

Supplement: Figure 6—source data 2. [file elife-92769-fig6-data2.zip › Figure 6-source data 2/Figure 6-source data 2/Figure 6-source data 2/Blots of Figure 6C.jpg]

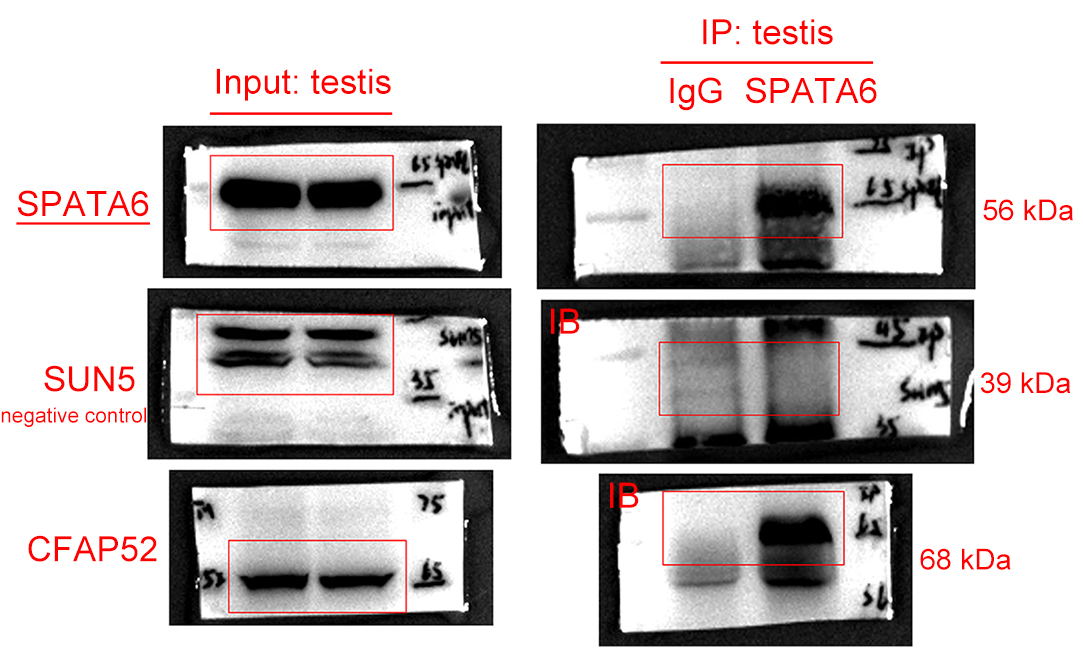

Supplement: Figure 6—source data 2. [file elife-92769-fig6-data2.zip › Figure 6-source data 2/Figure 6-source data 2/Figure 6-source data 2/Blots of Figure 6D.jpg]

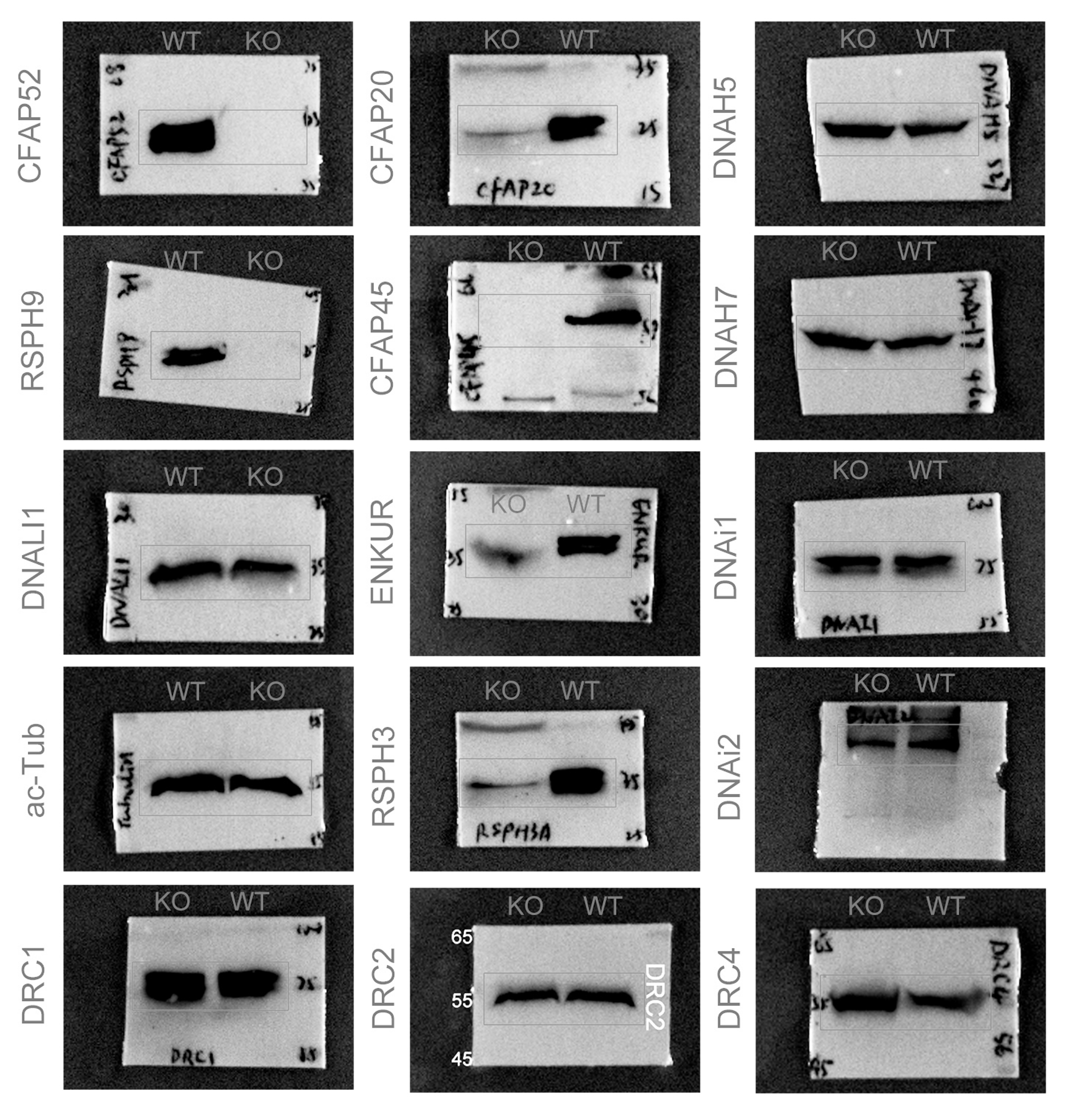

Supplement: Figure 7—source data 2. [file elife-92769-fig7-data2.zip › Figure 7-source data 2/Figure 7-source data 2/Blots for Figure 7A.jpg]

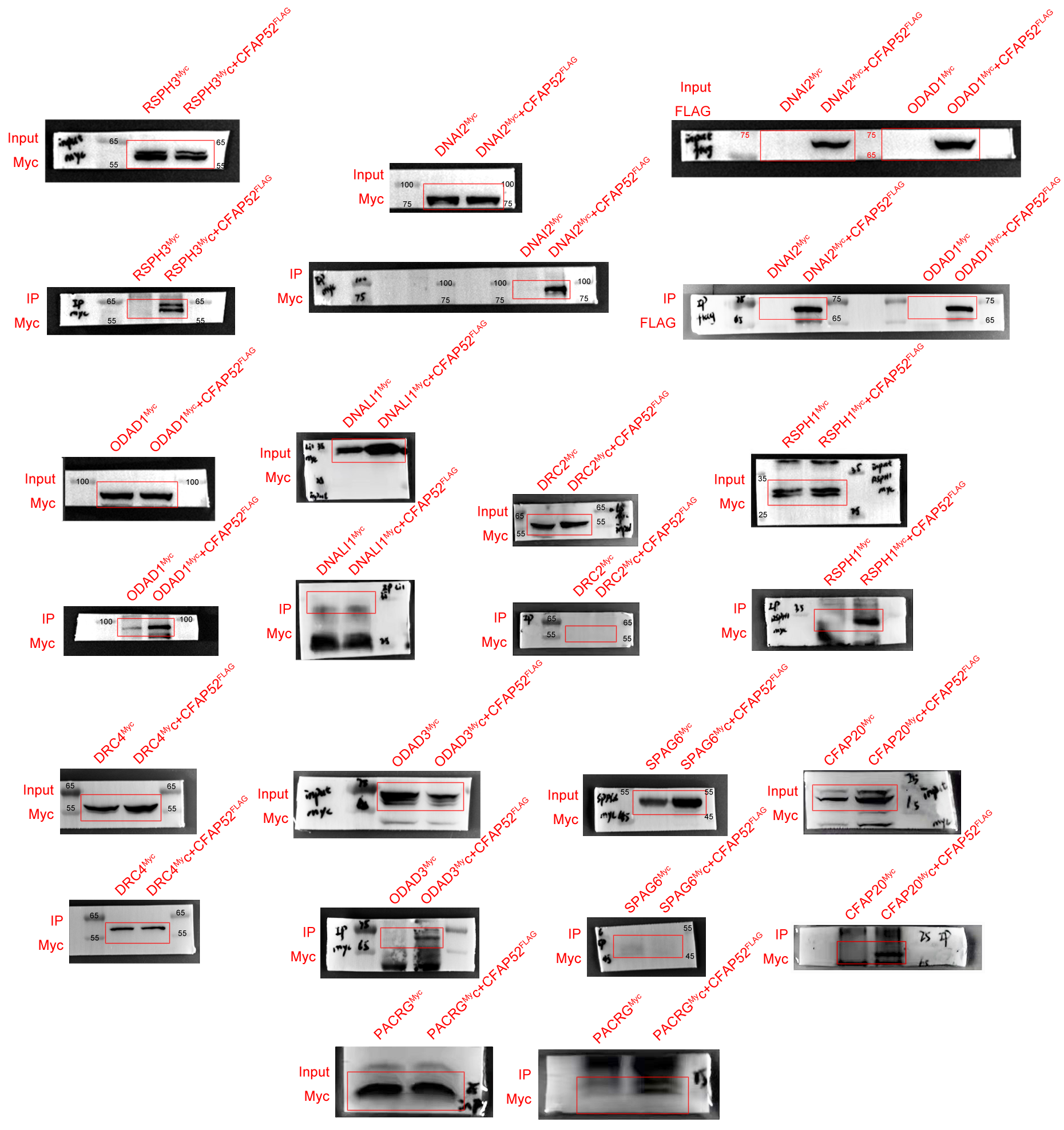

Supplement: Figure 7—figure supplement 1—source data 1. [file elife-92769-fig7-figsupp1-data1.zip › Figure 7-figure supplement 1/Figure 7-figure supplement 1/Figure 7-figure supplement 1.png]
