# Supplementary material for: Identification of CFAP52 as a novel diagnostic target of male infertility with defects of sperm head-tail connection and flagella development
Source: eLife. 2023 Dec 21;12:RP92769. doi: 10.7554/eLife.92769 (PMC10735225; doi:10.7554/eLife.92769)
Supplement: Figure 6—source data 1. [file elife-92769-fig6-data1.zip › Figure 6-source data 1/Figure 6-source data 1/Figure 6-source data 1.docx]

**Figure 6—source data 1.** Primers for plasmid construction.

| **Gene** | **Sequence (5’→3’)** |
| --- | --- |
| *SUN5* | CGGGGTACCCCGATGCCCCGGACGAGGAAC |
|  | TGCTCTAGAGCAGGATAGGG GCTCTAGGTGTGA |
| *SPATA6* | CGGGGTACCCCGATGCCGAAGGTGAAGGCGCTG |
|  | TTCAGTTGTTCAGCTGTCACTGGG |
| Mouse *CFAP52* | GGGGTACCCCATGGAAGAACAAGTTTTACCCGAGC |
|  | TGCTCTAGAGCACGAAGCAAATGGGTATTTCCACC |
| Human *CFAP52* | TACGGGATCACCAGTACTCGGGAGGCTGAGACAG |
|  | TCACCAGATATCTGGGGAACACAGACACTGGGGCC |
| *CFAP20* | GCCGCGATCGCCATGTTCAAGAACACGTTCCAGAGT |
|  | GTACGCGTTTGTTTGGCCTTGTTCTGAACTGG |
| *PACRG* | GCCGCGATCGCCATGCCGAAGAGGACTAAACTGCTGC |
|  | CGTACGCGTGTTCAGCAAGCACGACTCATAGGT |
| *DNAI2* | GGGGTACCCCATGGAGATCGTCTACGTGTACCTGA |
|  | TGCTCTAGAGCAGGTGGGCACATCCTCTTGCT |
| *ODAD1* | GGGGTACCCCATGATGGAGTTGGAAAGGCG |
|  | TGCTCTAGAGCAGTTGTAGCCCCTGGAGTCCTT |
| *ODAD3* | GGGGTACCCCATGACGTCCCCCCTGTGCTGG |
|  | CCGCTCGAGCGGAGATCTGCGCAACCTATTGCGCTTC |
| *RSPH1* | ATAAGAATGCGGCCGCTAAACTATATGTCGGACCTGGGCTCTGAGG |
|  | CCGCTCGAGCGGATCCTGGAGGTCTGACGGTTCTTCA |
| *RSPH3* | CCGCGATCGCCATGACAGACCGTAACCCTCGGAC |
|  | GTCGCGTCTCTGCCATAAGGTGTCCCCCCTCTT |
| *DNALI1* | GGGGTACCCCATGATACCCCCAGCAGACTCTC |
|  | TGCTCTAGAGCACTTCTTCGGTGCGATAATGC |
| *DRC2* | GGGGTACCCCATGTCTAAAAAAGGAAAAAAGCCC |
|  | TGCTCTAGAGCACAGGATATGGGAGGCAATGTG |
| *DRC4* | GGGGTACCCCATGGCACCCAAAAAGAAAGGGAAGA |
|  | CCGCTCGAGCGGTGTAGGGGCACCCACAAGTCCTG |
| *SPAG6* | GGGGTACCCCATGAGCCAGCGGCAGGT |
|  | CCGCTCGAGCGGGTTAATAAGAGGCTGATAGCTGTCG |
| *SPATA6*-N | GGGGTACCCCATGCCGAAGGTGAAGGCGCTG |
|  | TGCTCTAGAGCACAGATTTAAATGGGCCAGCCGC |
| *SPATA6*-C | GGGGTACCCCATGGGACCCTATGAATTCAAAAAGGAACAGA |
|  | TGCTCTAGAGCAGAAGCTTTCCTGTGTATGAGAAACAGAACTAC |
| *SPATA6*-M | GGGGTACCCCATGGAATGTCTGATAAGCTCAAGGAAGT |
|  | TGCTCTAGAGCACTTTGGTGTTGTTGAAGGTGC |
| *CFAP52*^ΔWD4~11^ | CGGGGTACCCCGATGGAAGAACAAGTTTTACCCGAGC |
|  | TGCTCTAGAGCATTGGCACTCGGTTGGCCAG |
| *CFAP52*^ΔWD9~11^ | GGGGTACCCCATGGAAGAACAAGTTTTACCCGAGC |
|  | TGCTCTAGAGCAAGCCTCCTCCAGCTTCTGAGTCT |
| *CFAP52*^ΔWD10~11^ | CGGGGTACCCCGATGGAAGAACAAGTTTTACCCGAGC |
|  | TGCTCTAGAGCAACCTTCCAATTCTCTGATTACTGT |
