# Supplementary material for: Identification of CFAP52 as a novel diagnostic target of male infertility with defects of sperm head-tail connection and flagella development
Source: eLife. 2023 Dec 21;12:RP92769. doi: 10.7554/eLife.92769 (PMC10735225; doi:10.7554/eLife.92769)
Supplement: Figure 7—source data 1. [file elife-92769-fig7-data1.zip › Figure 7-source data 1/Figure 7-source data 1/Figure 7-source data 1.docx]

**Figure 7—source data 1.** Antibodies used in this study.

| **Protein** | **Manufacturer** | **Cat No.** | **Usage** |
| --- | --- | --- | --- |
| CFAP52 | Cusabio | PA839781LA01HU | WB, IF |
| SUN5 | Proteintech | 17495-1-AP | WB, IF |
| SPATA6 | Proteintech | 11849-1-AP | WB, IP, IF |
| Acetylated-tubulin | Proteintech | 66200-1-Ig | WB, IF |
| PMFBP1 | Proteintech | 17061-1-AP | WB |
| SPATC1L | Novus | NBP2-01999 | WB |
| FAM46C | Proteintech | 25038-1-AP | WB |
| HOOK1 | Proteintech | 10871-1-AP | WB |
| ODF1 | Proteintech | 24736-1-AP | WB |
| IFT88 | Proteintech | 13967-1-AP | WB |
| CEP131 | Proteintech | 25735-1-AP | WB |
| CFAP45 | Mouse polyclonal (aa 280~551) | | WB, IF |
| ENKUR | Proteintech | 26440-1-AP | WB |
| RSPH3 | Proteintech | 17603-1-AP | WB |
| RSPH9 | Proteintech | 23253-1-AP | WB, IF |
| DNAH7 | Novus | NBP1-93613 | WB |
| DNAI1 | Proteintech | 12756-1-AP | WB, IF |
| DNAI2 | Proteintech | 17533-1-AP | WB |
| DNALI1 | Proteintech | 17601-1-AP | WB |
| DRC2 | Novus | NBP2-84617 | WB |
| DRC4 | Novus | NBP2-14037 | WB |
| β-actin | Abcam | ab8226 | WB |
| α-tubulin | Proteintech | 66031-1-Ig | IF |
| GAPDH | Proteintech | 60004-1-Ig | WB |
| Myc-Tag | Abmart | M20002 | WB, IP |
| Flag-Tag | Abmart | M20008 | WB, IP |
| Alexa Fluor 488-labeled Donkey Anti-Rabbit IgG | Beyotime | A0423 | IF |
| Alexa Fluor 555-labeled Donkey Anti-Mouse IgG | Beyotime | A0460 | IF |
| Goat anti-rabbit IgG-HRP | Abmart | M21002 | WB |
| Goat anti-mouse IgG HRP | Abmart | M21001 | WB |
| DAPI | Beyotime | C1002 | IF |
